# Parental Selective Reproduction: Genome-Editing and Maternal Behavior as a Potential Concern

**DOI:** 10.3389/fgene.2019.00532

**Published:** 2019-06-07

**Authors:** Andrea Lavazza

**Affiliations:** Centro Universitario Internazionale, Arezzo, Italy

**Keywords:** genetic engineering, social inequalities, ethics of reproductive medicine, genetic variety, human dignity and rights, eugenics

At the end of November 2018, Chinese geneticist He Jiankui declared he implanted embryos, that had been genetically modified with the CRISPR-Cas9 technique (Ran et al., 2013; Doudna and Charpentier, 2014), into two women. This announcement has aroused many comments and controversy both in public opinion and in the scientific community (e.g., Colata et al., 2018; Cyranoski and Ledford, 2018; Chadwick, 2019). As far as we know, this is the first time that a modification of the germline has been artificially and deliberately induced in two human beings, excluding mitochondrial replacement therapy (which is, however, aimed at preventing a specific type of genetic pathologies and does not allow for broad-spectrum interventions such as CRISPR-Cas9). In this case, He disabled a gene—CCR5—which is believed to play a role in allowing human immunodeficiency virus (HIV) to infect the cells.

The procedure has been the object of much criticism from the strictly medical-scientific point of view: among other things, it has been defined as “misguided, premature, unnecessary and largely useless” (Cyranoski, 2018). On the one hand, guaranteeing only partial genetic protection against HIV, which could also be obtained with other medical methods, the procedure, which has not yet been sufficiently tested, does not seem to compensate for the high risks it entails. On the other hand, the absence of the CCR5 gene can make lungs, liver, and brain more vulnerable to infections and chronic diseases other than HIV, starting with the flu (Falcón et al., 2005; Kohlmeier et al., 2008). In January 2019, He was suspended from his university—Southern University of Science and Technology of China, in Shenzhen—and placed under house arrest by the Chinese authorities. Meanwhile, the debate on the future of gene editing has become inflamed with different positions that, from the initial total rejection and request for sanctions, have expanded to include the simple wish of self-regulation on part of the scientific community (Akabayashi et al., 2018; Nie and Pickering, 2018; Hurlbut, 2019).

In particular, the World Health Organization has formed a panel of 18 scientific experts with the aim of setting international standards for the oversight of this practice, who proposed a global registry of studies related to human gene editing. And a group of 18 renowned scientists and bioethicists involved in CRISPR-Cas9 research has signed a 5-year global moratorium request “on all clinical uses of human germline editing” (Lander et al., 2019). Scholars believe that it is currently too risky to make genetically modified children (we are not yet able to hit the target accurately and we do not know what collateral consequences a local modification could produce). After the 5-year period, in which research is expected to make significant progress, there would be 2 years for each state to create public consensus on how to proceed. A legal ban is not requested, since it is thought to be sufficient to flag those who violate the moratorium, once the latter is adapted. Indeed, the Oviedo Convention on Human Rights and Biomedicine, ratified by 29 countries, states that (article 13), “An intervention seeking to modify the human genome may only be undertaken for preventive, diagnostic or therapeutic purposes and only if its aim is not to introduce any modification in the genome of any descendants.”

But even in the face of the highlighted dangers, the moratorium does not seem to be shared by all scientists working in the field (Cohen, 2019; Dzau et al., 2019). Some are skeptical about the usefulness of such a measure, which could have repercussions on research and funding. Leading geneticist George Church said it is only a matter of time before the genes of human embryos are “edited” to enhance their health and intelligence. And, to him, it is something we should embrace rather than fear (Cocker, 2019). Furthermore, it cannot be forgotten that the invitation to an appropriate use of the technology launched by the first International Summit on Human Gene Editing in December 2015 has been disobeyed. Not only did He edit embryos, but he also had the collaboration of other scientists who knew about the experiment and did not report it. Meanwhile, other pronouncements have been more “open-minded,” and interest in the possibility of human genetic enhancement has increased (cf. Lander et al., 2019).

## The Ethical Debate

From an ethical point of view, and in particular from the standpoint of the ethics of human reproductive genetic engineering (Liao, 2008), crossing this new threshold—i.e., the birth of the first genetically modified children—can lead to envisage both dystopian scenarios and more realistic, but not necessarily more positive, ones, above all due to the fact that any human germline editing has the potential to spread to the whole species with unpredictable consequences (the premise being that hypothesizing future situations is useful to evaluate possible choices to make, but that such predictions may be disproven).

Certainly, the possibility of easily modifying human DNA may cause the temptation to engineer major eugenic programs. The so-called rogue states could contemplate the—literal—creation of a new class of physically and cognitively enhanced individuals. Indeed, one might act on the gene that limits muscle development—as it has worked with sheep (Crispo et al., 2015). A bio-hacker has tried to do it but has apparently failed (Mosher et al., 2007; Lee, 2017). On the other hand, some genes may soon be identified that influence the development of intelligence (Plomin, 1999; Lavazza, 2018) as well as some psychological functions such as memory (Awasthi et al., 2019). Also, such rogue states might even want to create a society like that described by Huxley (1932/1998), where individuals are genetically engineered to be placed into predetermined classes based on intelligence and working abilities, some of them deprived of the dignity of human beings.

The only way to prevent these scenarios from occurring would probably be through preventive war or armed humanitarian interference (possibly under the guide of the UN), in order to protect (for the first time in human history) the population from very serious, large-scale scientific abuses—something that has already happened with the eugenic mass experiments conducted by the Nazi regime but without an international intervention (even though laws that imposed the sterilization of criminals and people deemed unfit to procreate were also introduced in many American states between the First and Second World War; cf. Kevles, 1985. And Germany itself was inspired by the laws of Oklahoma, cf. Bruinius, 2006). But if there was a serious threat of events of this type linked to a totalitarian eugenics, we could also evaluate the idea of placing a planetary ban on all gene-editing techniques, stopping research and making unavailable the necessary materials and tools, as is usually done in science (He Jiankui himself benefited from international collaboration).

This hypothesis obviously only makes sense from an ethical standpoint, because such a ban would be almost impossible to implement from a practical point of view. And there would also be good reasons to oppose a global research ban, because a technique like CRISPR-Cas9 has opened extraordinary treatment opportunities and to renounce it, preventing many people from avoiding a fatal disease due to potential risks on other fronts, would seem unreasonable and immoral. An important *technical* distinction in fact is that between genetic correction and genetic enhancement, where genetic correction means the correction of rare mutations with high probability of causing a severe single-gene disease, while genetic enhancement means the generic attempt to improve individuals and the whole species.

Scientific knowledge and technological tools are often ambivalent and can be used against shared goals and values. This requires that there be a rational ethical reflection and an informed public debate with a consequent action aimed at avoiding undesirable outcomes through the procedures of liberal democracy. Here probably comes the most difficult point, because the main risks related to the diffusion of genome-editing techniques do not seem to come from totalitarian eugenics (which seems improbable), but rather from liberal eugenics, as it has been defined (cf. Habermas, 2003). Liberal eugenics implies that the decisions about reproductive selection should be a) voluntary, i.e., taken without coercion; b) individualistic, i.e., conducted by individual families and for individual children, not imposed by the state or for specific racial groups or gene pools; and c) state-neutral, i.e., pursued by parents without the state promoting any particular goal about the people who should be born.

Indeed, if genome editing were to become widely available, it would probably not be the state to impose on citizens how to modify their offspring for a collective purpose set by the state itself: families themselves would freely decide if and how to modify the DNA of their children. Consider what He said in November 2018 at the Second International Summit on Human Genome Editing in Hong Kong: “Do you see your friends or relatives who may have a disease? They need help. For millions of families with inherited disease or infectious disease, if we have this technology, we can help them” (Cyranoski, 2018). What parent would not do anything for her child to be in good health and successful?

Now, it is not a question of relying on rhetorical or generic statements to develop an *ad hoc* argument: the point is to take a relatively realistic scenario as the object of specific philosophical reflection. The principle in question, about procreative decision making, was formulated by Savulescu: the so-called principle of Procreative Beneficence. According to it, “couples (or single reproducers) should select the child, of the possible children they could have, who is expected to have the best life, or at least as good a life as the others, based on the relevant, available information” (Savulescu, 2001, p. 415). In the perfectionist view, since the distinction between embryo selection and modification is normatively relevant (Liao, 2019), that principle should be completed with the “moral obligation to create children with the best chance of the best life” (cf. Harris, 2007; Savulescu and Kahane, 2009).

While it is difficult to challenge the use of genome editing for the prevention or treatment of diseases that endanger the life of the unborn child, on the other hand, it is well-known that the line between cure and enhancement—along the continuum that goes from the treatment of serious pathologies to interventions of physical or cognitive cosmesis—is very blurred and any attempt to trace it clearly does not find easy consensus.

Compared to protection from an almost certain transmission of HIV, how should one evaluate the attempt to, say, genome-edit the DNA inside the father's sperm cells with the aim of reducing the child's risk of developing Alzheimer's disease (AD), knowing that it is uncertain what the genetic component of AD is (Regalado, 2018)? Therefore, it makes sense to imagine that reproducers, faced with the possibility of modifying the genetic makeup of their children, will try to obtain the best for them, in terms of both physical health and human flowering understood in the broader sense of the expression.

An influential, and here relevant, ethical–philosophical normative line of argument is the “procreative liberty” theory, supported by Robertson (1983, 1994). The basic idea is that unless the state has very good reasons not to allow it, people should be free to have access to all the technologies provided by reproductive medicine to have a child with specific traits. Given that the freedom to have sex without reproduction is recognized by the right to access birth control and abortion (at least in the United States), Robertson advocates a constitutional right “to become pregnant and to parent” in such a way as to have “the freedom to reproduce when, with whom, and by what means one chooses” (Robertson, 1983). Procreative liberty implies the right of future parents to not suffer any interference from the state and this freedom also concerns the external conception and all the technological means involved in procreation.

The point is that if people are free to choose whether they reproduce or not, and if the genetic characteristics of the expected offspring influence that decision, then parents should be free to make a prenatal selection of the characteristics of their children. And to guarantee this right, which, according to Robertson, should be constitutional, no prohibition should be placed on the parents' desire to have a child with certain characteristics, and if a trait turns out to be decisive for the parents' choice to reproduce or not, then the decision to engineer this trait should receive legal protection as an exercise of procreative liberty. Robertson speaks of public restraints, but other jurists have tried to extend the same arguments about procreative freedom to the private sector and to medical professionals (Fox, 2018).

## New Objections To Procreative Liberty

Now, many objections can be raised against the principles of Procreative Beneficence and Procreative Liberty. One type of objection concerns the protection of the unborn child, whose moral status is certainly a matter of discussion, but which should not be completely overlooked in the balance of rights (Marquis, 1989). Another type of objection states that “parents may misjudge the best features for their children's lives and that therefore it is preferable to act with caution, especially when it comes to genetic editing. Other objections refer to the children's right not to be burdened with their parents' expectations, as there is an asymmetric power relation in place by which parents may decide for their children that they should be particularly competitive in some areas over others” (Lavazza, 2018).

This will be unlikely to discourage reproducers, if given the chance to do so, from trying in every way to give their children what they think will be the best tools for a satisfying and happy life, including modifications of DNA to prevent diseases, slow down aging, enhance physical endurance, or cognitive abilities. Is it not typical of parents, and of mothers especially, to endure every sacrifice and to try in every way to secure the best for their children? I believe that it is precisely this well-meaning type of eugenics that is to be feared most, as it carries with it many risks that should be immediately taken into serious consideration. In fact, this would be a liberal eugenics that would hardly be opposed, at least in its early stages, precisely because it seems oriented to the welfare of a future individual while not harming anyone else.

However, there are some strong reasons for concern. Firstly, as was emphasized in He's case, security reasons require great caution in intervening on the genome. We do not know what the long-term consequences may be or what interactions may happen between induced mutations and random mutations in future generations (as the editing would affect the germline), and we cannot experiment on human beings to acquire the knowledge that we lack today. Secondly, even if the problem of safety were solved, children have the right to autonomy, and they might not share the idea of a modified custom genome that their parents would choose for them. In this sense, the principle of Procreative Beneficence and the procreative liberty should be mitigated by the principle of *the unborn's right to an “open future”* not conditioned by their parents' eugenic choices. This principle would have consequences on the permissibility of genome editing. On the basis of this (provided this is technically possible), one may allow interventions aimed at giving a general broad-spectrum advantage, such as intelligence, but not modifications that would affect the child's existential path, such as above-average muscle development or a specifically enhanced sense like hearing (the topic is very complex, though, and cannot be exhausted in a contraposition between principles, cf. Glover, 2006; Gheaus, 2017).

Thirdly, there is a social reason in the broad sense. We do not know what wide composition effects may arise if every parent could have a genetically modified child. A well-known example is the choice of gender: if every family only wanted male children, based on the view that life for males is usually easier in society, this would result—contrary to individual expectations—in a worse condition for all, with a lack of women and a very strong competition between men. Also, gene editing might produce too many people with inclinations or skills of a certain type, impoverishing society of other talents that could suddenly become necessary. Not to mention the potential reduction of genetic variety (since modifications could converge on a few preferred traits), variety being one of the tools with which evolution makes the species prosper, as the more polymorphisms are found in the population, the more easily there may be some individual capable of withstanding new environmental challenges, be it a bacterial or viral threat, harsh climatic conditions, and so forth.

Moreover, inequality could also get worse insofar as only some reproducers would likely have access to the most advanced forms of genetic editing, which would make their children much more advantaged and likely to occupy the pre-eminent positions in the economic, political, and cultural hierarchy. A scenario that even Hawking (2018) feared. His worry was that richer social groups would genetically modify their children to create a superhuman race with enhanced cognitive abilities, resistance to disease, and quasi-immortality, dividing humanity into genetic “haves” and “have-nots.” This is linked to a further concern, namely, the possible lesser acceptance and tolerance of both physical and mental differences. We have only recently succeeded in introducing the concept of “neurodiversity” to refer to what was previously called a disease or even constituted a stigma, and with gene editing, we could go back to considering individuals affected by them as marked by “lesser genetic quality.” This might lead to discrimination or even to the will to eliminate such people prior to birth, if they cannot be genetically modified. But when it comes to people with severe disabilities, it is not possible to know what life they may have and what contribution they may be able to give to society, as Hawking's case shows. And this calls for further caution.

Furthermore, it is possible that scientific progress will make increasingly refined gene editing techniques available. In this way, there would be more and more high-performing individuals, with each cohort of newborns more efficient than the previous one. It could thus create a stratification of more or less efficient individuals. In this case, it would be more difficult to guarantee fairness and equality of treatment.

## Conclusion

A group of British experts, just before He's experiments, has cautiously endorsed genetic editing interventions on embryos, considering such interventions morally permissible so long as they do not contradict a clear principle: “the use of gametes or embryos that have been subject to genome editing procedures (or that are derived from cells that have been subject to such procedures) should be permitted only in circumstances in which it cannot reasonably be expected to produce or exacerbate social division or the unmitigated marginalization or disadvantage of groups within society” (Nuffield Council on Bioethics, 2018: XVII). In my opinion, the principle should be more restrictive, to avoid all the risks related to parental eugenics that I have listed above.

In fact, regardless of a possible moratorium—which does not seem to be on the horizon—the permissibility of human germline editing is being pursued both by a part of the scientific community and by a juridical–normative approach to reproduction that gives legal or biological parents the maximum freedom of choice. But the introduction of the CRISPR-Cas9 technique, which makes it easier than ever before to intervene on DNA, has changed the scenario, because individual reproductive liberty risks reflecting with both predictable composition effects and unpredictable consequences on the entire human species.

Accordingly, doctors and scientists, as well as political and health authorities, should take care not to surrender to the understandable claims of the parents and allow genome-editing on embryos only in a few well-defined cases that involve the risk of death or of extremely disabling life conditions. A day may come when we can all be genetically improved without incurring all the risks mentioned above. Only then I believe that it will be fair to do so.

## Author Contributions

The author confirms being the sole contributor of this work and has approved it for publication.

### Conflict of Interest Statement

The author declares that the research was conducted in the absence of any commercial or financial relationships that could be construed as a potential conflict of interest.
